# Using glycyrrhizic acid to target sumoylation processes during Epstein-Barr virus latency

**DOI:** 10.1371/journal.pone.0217578

**Published:** 2019-05-24

**Authors:** Gretchen L. Bentz, Angela J. Lowrey, Dustin C. Horne, Vy Nguyen, Austin R. Satterfield, Tabithia D. Ross, Abigail E. Harrod, Olga N. Uchakina, Robert J. McKallip

**Affiliations:** Division of Biomedical Sciences, Mercer University School of Medicine, Macon, Georgia, United States of America; University of Nebraska-Lincoln, UNITED STATES

## Abstract

Cellular sumoylation processes are proposed targets for anti-viral and anti-cancer therapies. We reported that Epstein-Barr virus (EBV) latent membrane protein 1 (LMP1) dysregulates cellular sumoylation processes, contributing to its oncogenic potential in EBV-associated malignancies. Ginkgolic acid and anacardic acid, known inhibitors of sumoylation, inhibit LMP1-induced protein sumoylation; however, both drugs have adverse effects in hosts. Here we test the effects of glycyrrhizic acid, a medicinal botanical extract with anti-inflammatory, anti-carcinogenic, and anti-viral properties, on cellular sumoylation processes. While glycyrrhizic acid is known to inhibit EBV penetration, its affect on cellular sumoylation processes remains to be documented. We hypothesized that glycyrrhizic acid inhibits cellular sumoylation processes and may be a viable treatment for Epstein-Barr virus-associated malignancies. Results showed that glycyrrhizic acid inhibited sumoylation processes (without affecting ubiquitination processes), limited cell growth, and induced apoptosis in multiple cell lines. Similar to ginkgolic acid; glycyrrhizic acid targeted the first step of the sumoylation process and resulted in low levels of spontaneous EBV reactivation. Glycyrrhizic acid did not affect induced reactivation of the virus, but the presence of the extract did reduce the ability of the produced virus to infect additional cells. Therefore, we propose that glycyrrhizic acid may be a potential therapeutic drug to augment the treatment of EBV-associated lymphoid malignancies.

## Introduction

Protein post-translational modifications, such as ubiquitination and phosphorylation, allow cells to respond to both external and internal stimuli and are vital to numerous cellular events. The modification of proteins by the small ubiquitin-like modifier or SUMO was identified in 1997 [1]. There are four characterized human SUMO isoforms (SUMO-1, -2, -3, and -4), and SUMO-1 and SUMO-2/3 are ubiquitously expressed in the body. Protein sumoylation is similar to ubiquitination in that it is a dynamic, multi-step process. First, the translated SUMO-pro-peptide undergoes maturation [2–5]. Second, matured SUMO is activated in an ATP-dependent manner by the SUMO-activating enzyme [2–5]. Third, the SUMO-conjugating enzyme, Ubc9, recognizes the conserved sumoylation motif (ΨKxD/E motif, where Ψ represents a hydrophobic amino acid) within the target protein and mediates the formation of an isopeptide bond with the activated protein and the lysine residue within the SUMO motif of the target protein [2–6]. De-sumoylation of the target protein is mediated by sentrin-specific proteases or SENPs [7].

At any given time, only a small percentage of a population of a target protein is found in its sumoylated form; however, the effect of sumoylation on the target protein can be long-lasting [8]. The post-translational modification of a protein by SUMO can modulate a protein’s function in various ways, including its localization, its turnover, and its ability to interact with other proteins or DNA [6,9,10]. The end result is the modulation of numerous cellular processes, such as nuclear trafficking, cell division, DNA replication, DNA damage responses, transcription, and chromosome segregation [11–17]. Understandably, dysregulation of sumoylation processes are a feature of a variety of types of cancer [2,18–20].

Because sumoylation processes appear to modulate tumorigenesis, members of the SUMO machinery have been proposed as potential targets for anti-cancer therapies [2,21]. The most common target is the SUMO-conjugating enzyme, Ubc9, where sumoylation processes can be inhibited by knockdown of Ubc9 or over-expression of an enzymatically inactive Ubc9 (Ubc9 C93S) [21]. In addition, the antibiotic Spectomycin B1 can bind directly to Ubc9, inhibiting the formation of the Ubc9-SUMO intermediate [22]; however, the availability of this antibiotic is highly limited. There is only one known SUMO-activating enzyme, which is a heterodimer of SAE1 and SAE2, so regulating its activity or expression can also modulate sumoylation processes. Interestingly, the botanical extracts ginkgolic acid (an alkylphenol from *Ginko biloba*), anacardic acid (a structural analog of ginkgolic acid), and davidiin (an ellagitannin from *Davidia involucrata*) bind to the SUMO-activating enzyme (SAE1/2) and impair it from interacting with and activating the mature SUMO [23,24]. While there are additional cellular targets for these drugs, their ability to inhibit sumoylation processes has been documented [23–25]. However, these extracts can be toxic and allergenic at therapeutic doses [26–31]. In the current study we determined if a fourth, less toxic, botanical extract, specifically glycyrrhizic acid, could also target the sumoylation process.

Glycyrrhizic acid is a triterpene from licorice root (*Glycyrrhiza glabra* in southern Europe and *Glycyrrhiza uralensis* in east Asia) [32,33], which has been used for traditional medicinal purposes for almost two thousand years. The most common use for glycyrrhizic acid is to treat liver disease due to the ability of the drug to inhibit liver fibrosis, steatosis, and necrosis as well as promote cell regeneration [34]. Glycyrrhizic acid is also reported to have anti-inflammatory, anti-carcinogenic, and anti-viral properties [32,33,35,36]. Of specific interest to our lab, glycyrrhizic acid has been shown to have anti-viral activity to members of the *Herpesviridae* family including Epstein-Barr Virus (EBV) [35,37–55].

Following an initial lytic infection, the linear viral genomes circularize, forming episomes, and establishing life-long latent infections in hosts. Periodically, the latent virus undergoes reactivation, resulting in the production and release of new infectious virus. EBV establishes a life-long latent infection in over 90% of the world’s population. Latent EBV infections are associated with distinct lymphoid malignancies, including post-transplant lymphoproliferative disorder (PTLD), and AIDS-associated CNS lymphomas [56,57]. These malignancies are characterized as Type III EBV latency, which is also observed in the laboratory in lymphoblastoid cell lines (LCLs) that are established by EBV-mediated transformation of naïve B-cells and exhibit sustained cellular proliferation and survival due to the constitutive activation of cellular signaling pathways.

The principal viral oncoprotein implicated in these EBV-associated malignancies is Latent Membrane Protein (LMP)-1, a constitutively activated integral membrane signaling protein that mimics the tumor necrosis factor receptor family members, such as CD40 [58]. LMP1 activates multiple signal transduction events through its extensively characterized C-terminal activating regions, CTAR1 and CTAR2 [58–61]. We identified the first function for the less studied CTAR3 in its ability to hijack the SUMO-conjugating enzyme and increase the sumoylation of cellular proteins [62]. Our recent work documented that LMP1 also induced the *sumo* promoters, increasing the intracellular pools of SUMO available for protein prost-translational modifications [63]. Together, our findings suggest that LMP1 dysregulates cellular sumoylation processes in order to maintain viral latency, modulate innate immune responses, and control oncogenesis [25,62,64].

Glycyrrhizic acid has been proposed to interrupt herpesvirus latency [38,39]. Because we recently identified a role for sumoylation processes in the maintenance of Epstein-Barr virus (a ubiquitous human γ-herpesirus) latency [25], we were interested in determining if one mechanism by which glycyrrhizic acid interrupts herpesvirus latency is by inhibition of cellular sumoylation processes. We show here that glycyrrhizic acid, a botanical extract often used for medicinal purposes, inhibits endogenous sumoylation processes in EBV-transformed LCLs. These findings suggest that glycyrrhizic acid inhibits the SUMO machinery from interacting with SUMO. In addition, treatment with glycyrrhizic acid induced very low levels of EBV reactivation. Interestingly, the extract did not affect viral replication in ZTA-induced cells, but as previously documented [46], the presence of glycyrrhizic acid decreased the capability of new virus to infect new cells. Therefore, we propose that treatment with glycyrrhizic acid may be beneficial in the treatment of EBV-associated malignancies as well as other diseases in which sumoylation processes are up-regulated.

## Materials and methods

### Cells

Human embryonic kidney (HEK) 293 cells, paired BL41 cells, and Raji cells were maintained as previously described [25,62–64]. EBV-transformed LCLs were generated by the Lineberger Comprehensive Cancer Center Tissue Culture Facility and cultured in RPMI with 10% FBS. 293 EBV WT cells were a gift from Dr. Wolfgang Hammerschmidt (Munich, Germany) and maintained as previously described [25,65].

### Plasmids/siRNA

Flag-LMP1 expression constructs have been described previously [61,66]. GFP-KAP1 was purchased from Addgene. The BZLF1-expressing plasmid was a gift from Dr. Wolfgang Hammerschmidt [65].

### Immunoprecipitation (Native)

Transfections were performed as previously described [25,64]. 48 hours post-transfection cells were harvested, washed with PBS, and lysed in 1mL cold cell lysis buffer (RIPA; 20 mM Tris pH 7.5, 150 mM NaCl, 1% Igepal, 0.5% sodium deoxycholate, 0.1% sodium dodecyl sulfate/SDS, 1 mM ethylenediaminetetraacetic acid/EDTA) containing DNase I, benzonase, and EDTA-free protease inhibitors. Following addition of lysis buffer, cells were further disrupted via a series of four freeze-thaw cycles, and supernatant fluids were collected after centrifugation at 7500 x g. Supernatant fluids were then incubated with 1 ug of antibody overnight at 4°C. Magnetic Protein G beads (Life Technologies) were added to the samples, which were then incubated 4–6 hours at 4°C. Beads were washed four times with cell lysis buffer and resuspended in 4x Laemmli (BioRad) loading buffer.

### *In vitro* sumoylation

*In vitro* SUMOylation was performed using the SUMO1 Conjugation Kit from Boston Biochem (K-710). The assay was accomplished with 10μM purified substrate protein UBE2K/E2-25K (SP-200; Boston Biochem) according to the assay protocol provided with the conjugation kit. Prior to addition of ATP, which triggers SUMOylation reaction, samples were treated with 20μM ginkgolic acid or 3mM glycyrrhizic acid with one reaction left untreated as a control. For each reaction, a second reaction without ATP was performed as a negative control. Reactions were incubated at 37°C for 60 minutes, and then 5X SDS buffer containing DTT was added and the samples were incubated 5 minutes at 90°C. SDS-PAGE was performed and the gel stained in Coomassie blue overnight. The gel was then destained in 15% isopropanol and 10% acetic acid for 2 hours before being placed in water and imaged using the ChemiDoc Touch Imaging System (BioRad).

### Western blot analysis

Western blot analyses were performed as previously described [25,62,64,67], with the exception that samples were transferred to polyvinylidene fluoride membranes (PVDF) using the TransBlot Turbo Transfer System (BioRad). Following staining and washing with the appropriate primary and horseradish peroxidase-conjugated secondary antibodies, bands were visualized with enhanced chemiluminescence (ECL; Advansta) reagent using the ChemiDoc Touch Imaging System (BioRad).

### Viral induction by ZTA

293 EBV WT were induced by transfection of ZTA-expression plasmids. Cells and supernatant fluids were collected 48 hours after transfection. Total DNA was isolated from cells and supernatant fluids as previously described [25]. The remaining supernatant fluids were added to Raji cells. 72 hours later the percentages of GFP-positive Raji cells and the number of GFP-positive Raji cells per field of view were determined by immunofluorescence microscopy [68].

### Real-time PCR

DNase-resistant encapsidated virion associated DNA was harvested and qPCR performed for *gapdh* and EBV W-1 using the Bio-Rad Universal SYBR Green Supermix (Bio-Rad) as previously described [25,69–71]. Samples and experiments were run in triplicate.

### Treatment of Cells

Ginkgolic acid C15:1 was purchased from Sigma and glycyrrhizic acid was purchased from Spectrum Chemical Manufacturing Corporation. Cells were treated with varying concentrations of glycyrrhizic acid (0 mM, 0.5 mM, 1 mM, 2 mM, 3 mM and 4 mM). In some experiments, cells were either treated with DMSO (vehicle control) or 25 μM ginkgolic acid.

### Antibodies

Anti-GAPDH (FL-335), anti-PARP (F-2), anti-Ubiquitin (A-5), anti-Myc (9E10), and anti-caspase 3 (E-8) antibodies were purchased from Santa Cruz. Anti-SENP2 (ab131637), anti-PIAS1 (EPR2581Y), anti-RanBP2 (ab64276), anti-SAE1 (EPR15398), anti-SUMO-1 (EP298), and anti-SUMO-2/3 (ab233222) antibodies were purchased from Abcam. Anti-SAE2 (SAB3500487) and anti-UBC9 (SAB1309192) antibodies were purchased from Sigma.

### Statistical analysis

Statistical analyses were performed using the unpaired, two-tailed, Student’s T-test. Data are presented as means ± the standard deviation for samples run in triplicate and independent experiments performed in triplicate. Differences were considered statistically significant when P-values were less than 0.05.

## Results

### Glycyrrhizic acid decreased levels of sumoylated proteins in a dose-dependent manner

**Fig 1:**
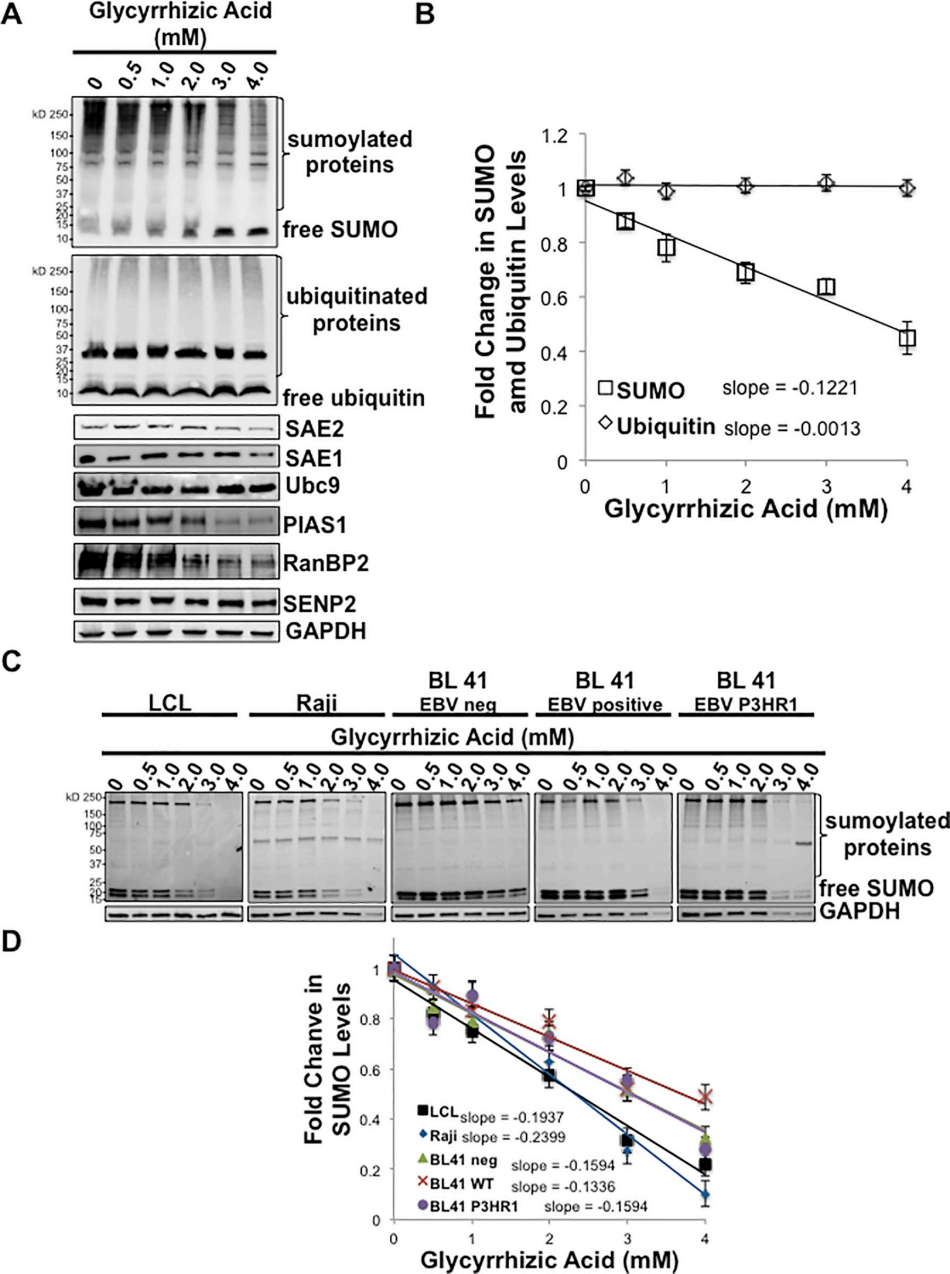
Glycyrrhizic acid decreased levels of sumoylated proteins in a dose-dependent manner. A-B) LMP1-expressing HEK 293 cells were treated with graduated doses of glycyrrhizic acid. 48 hours post-treatment, cells were harvested, lysed, denatured, and A) Western blot analyses performed to detect SUMO-1/2/3, SAE1, SAE2, Ubc9, PIAS1, RanBP2, SENP2, and Ubiquitin levels. GAPDH was used as a loading control. B) Densitometric analysis of repeat experiments was performed to determine relative SUMO and relative Ubiquitin levels. Results are shown as the means ± the standard deviation of experiments performed in triplicate. C) EBV-transformed lymphoblastoid cell lines (LCLs), Raji cells, EBV-negative BL 41 cells, EBV-positive BL-41 cells, and BL41 cells infected with a mutant strain of EBV (P3HR1) were treated with graduated doses of glycyrrhizic acid. 24 hours post-treatment, cells were harvested, lysed, denatured, and Western blot analyses performed to detect SUMO-1/2/3 levels. GAPDH was used as a loading control. D) Densitometric analysis of repeat experiments was performed to determine relative SUMO. Results are shown as the means ± the standard deviation of experiments performed in triplicate.

To begin our investigation into the effect of glycyrrhizic acid on sumoylation levels, LMP1-expressing HEK 293 cells were treated with graduated amounts of glycyrrhizic acid Results showed that as glycyrrhizic acid levels increased, levels of sumoylated proteins, depicted by the laddering of slower migrating bands, decreased (Fig 1A). Levels of free SUMO (~12 kDa) increased as glycyrrhizic acid levels increased (Fig 1A), which suggested that sumoylation processes were inhibited resulting in the accumulation of free SUMO. Densitometric analysis of repeat experiments revealed that glycyrrhizic acid treatment resulted in a consistent dose-dependent decrease in levels of sumoylated proteins (Fig 1B).

Because of the similarities between sumoylation processes and ubiquitination processes [6,72], the effect of glycyrrhizic acid on levels of ubiquitinated proteins was also analyzed. Western blot analyses (Fig 1A) and densitometric analysis of repeat experiments (Fig 1B) showed no significant changes in levels of free ubiquitin (~9 kDa) or ubiquitinated proteins (laddering of slower migrating bands) following treatment with glycyrrhizic acid treatment. Together these data demonstrate that glycyrrhizic acid can inhibit cellular sumoylation processes without affecting ubiquitination processes.

To determine if the detected decrease in levels of sumoylated proteins was due to loss of the SUMO machinery, Western blot analyses were performed to detect specific members of the SUMO machinery for each step of the sumoylation process (Fig 1A). Findings showed that treatment with glycyrrhizic acid did not have any effect on endogenous levels of the SUMO-activating enzyme (the dimer of SAE1 and SAE2), the SUMO-conjugating enzyme (Ubc9), or the de-sumoylating enzymes (SENP2). Interestingly, higher levels of treatment with glycyrrhizic acid did result in decreased levels of E3 SUMO-ligases (PIAS1 and RanBP2). These results suggest that glycyrrhizic acid can modulate the expression of the SUMO-ligases but does not have an effect on the expression of the remaining SUMO machinery.

To establish if glycyrrhizic acid could also modulate levels of sumoylated proteins in B-cells, we used five different B-cell lines. EBV-transformed naïve B-cells (a lymphoblastoid cell line previously established from an unidentified donor; LCLs), Raji cells, and paired BL41 cell lines (EBV negative, EBV positive, or infected with a mutant EBV, P3HR1, that has a deletion of EBNA2 resulting in loss of LMP1 expression [73–75]) were treated with graduated amounts of glycyrrhizic acid. Results showed that as glycyrrhizic acid levels increased, levels of sumoylated proteins decreased in all B-cell lines (Fig 1C). Densitometric analysis of repeat experiments revealed that no significant changes in the decreased levels of sumoylated proteins occurred when comparing the different B-cell lines (Fig 1B). These findings led us to propose that glycyrrhizic acid inhibits sumoylation processes.

### Glycyrrhizic acid affects LCL growth and survival

**Fig 2:**
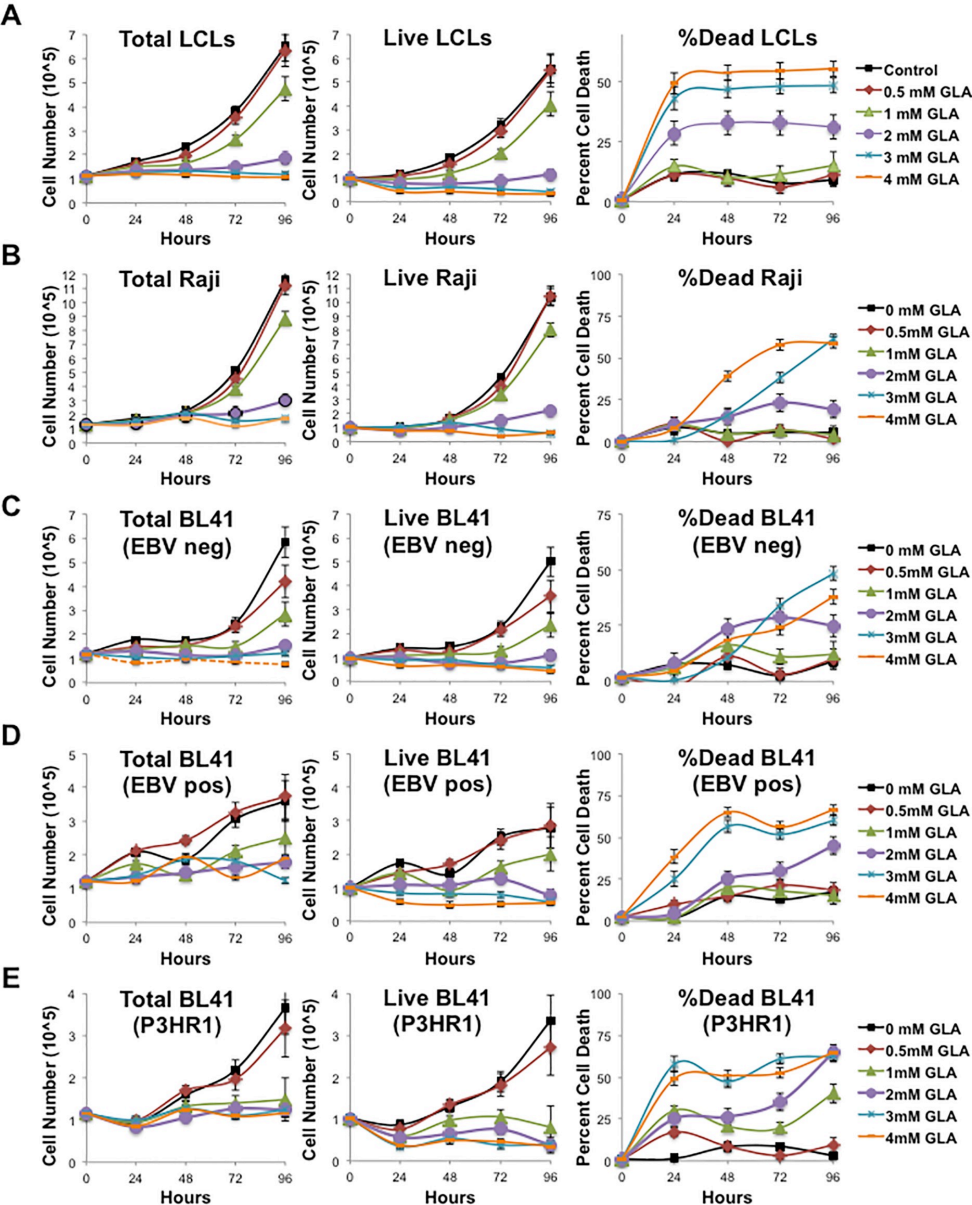
Glycyrrhizic acid affected growth and survival. **A)** EBV-transformed LCLs, **B)** Raji cells, **C)** EBV-negative BL 41 cells, **D)** EBV-positive BL 41 cells, and **E)** P3HR1-infected BL 41 cells were treated with graduated doses of glycyrrhizic acid and total cell number (left), live cell number (middle), percent cell death (right) were determined. Results are shown as the means ± the standard deviation of a representative experiment of samples run in triplicate and experiments performed in triplicate.

EBV-transformed LCLs exhibit sustained cellular proliferation and survival due to the constitutive activation of LMP1. Sumoylation processes have been documented to help regulate cell division and cell survival [11–17], so cell growth and survival was analyzed. Total cell number, live cell number, and the percent cell death were quantitated by Trypan Blue exclusion for LCLs (Fig 2A), Raji cells (Fig 2B), EBV-negative BL 41 cells (Fig 2C), EBV-positive BL41 cells (Fig 2D), and P3HR1-infected BL 41 cells (Fig 2E) treated with graduated doses of glycyrrhizic acid. Results showed that control-treated B-cells and B-cells treated with 0.5 mM glycyrrhizic acid exhibited similar growth and death curves. B-cells treated with 1 mM glycyrrhizic acid exhibited a slight lag in cell growth, but no significant change in cell death when compared with control-treated B-cells. Treatment of B-cells with 2, 3, or 4 mM of glycyrrhizic acid inhibited cell growth and significantly (P < 0.05) increased cell death by 96 hours post-treatment when compared with their control-treated counterparts. These findings suggest that glycyrrhizic acid can inhibit B-cell growth and induce B-cell death.

### Higher doses of glycyrrhizic acid induce apoptosis

**Fig 3:**
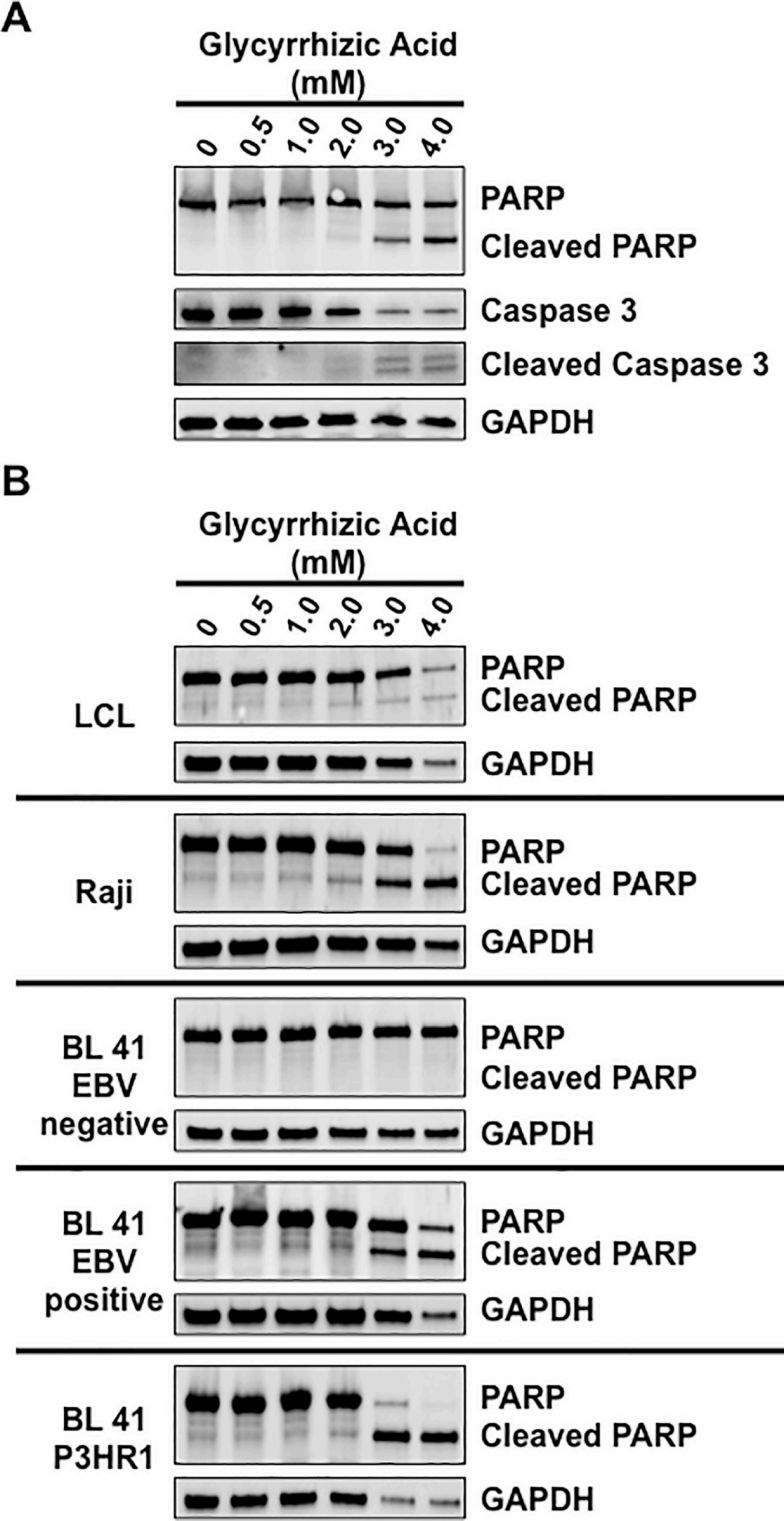
Higher doses of glycyrrhizic acid induced LCL apoptosis. **A)** LMP1-expressing HEK 293 cells and **B)** LCLs, Raji cells, BL 41 EBV negative cells, BL 41 EBV positive cells, and BL 41 P3HR1 cells were treated with graduated doses of glycyrrhizic acid. 48 hours post-treatment, cells were harvested, lysed, denatured, and Western blot analyses performed to detect total and cleaved PARP, caspase-3, or GAPDH.

To further analyze the effect of glycyrrhizic acid on cells, Western blot analyses were performed on lysates from LMP1-expressing HEK 293 cells treated with graduated doses of glycyrrhizic acid to detect the cleavage of poly (ADP-ribose) polymerase (PARP) and caspase 3, which occurs during apoptosis. Data showed that in control-, 0.5 mM-, and 1 mM-treated cells cleaved PARP and cleaved caspase 3 were not detected (Fig 3A). Increasing levels of cleaved PARP and cleaved caspase 3 and decreased levels of un-cleaved PARP and un-cleaved caspase 3 were detected when cells were treated with 2, 3, or 4 mM of glycyrrhizic acid. Similar experiments were done on a collection of B-cell lines (Fig 3B), and data confirmed that cleaved PARP was readily detectable in LCLs, Raji cells, BL 41 EBV positive cells, and BL 41 P3HR1 cells treated with 3.0 or 4.0 mM glycyrrhizic acid. The cleavage of PARP was not detected in BL 41 EBV negative cells. These data suggest that higher doses of glycyrrhizic acid can induce apoptosis in HEK 293 cells and B-cells, but increased apoptosis is observed in EBV-positive B-cells when compared with EBV-negative B-cells. While the mechanism behind the increased PARP cleavage in the EBV-positive cells is unknown, these findings led us to propose that 2 mM of glycyrrhizic acid was the optimal dose of glycyrrhizic acid to inhibit cellular sumoylation processes in EBV-transformed B-cells, inhibiting cell growth, with modest induction of cell death.

### Glycyrrhizic acid inhibited SUMO from interacting with the sumoylation machinery

**Fig 4:**
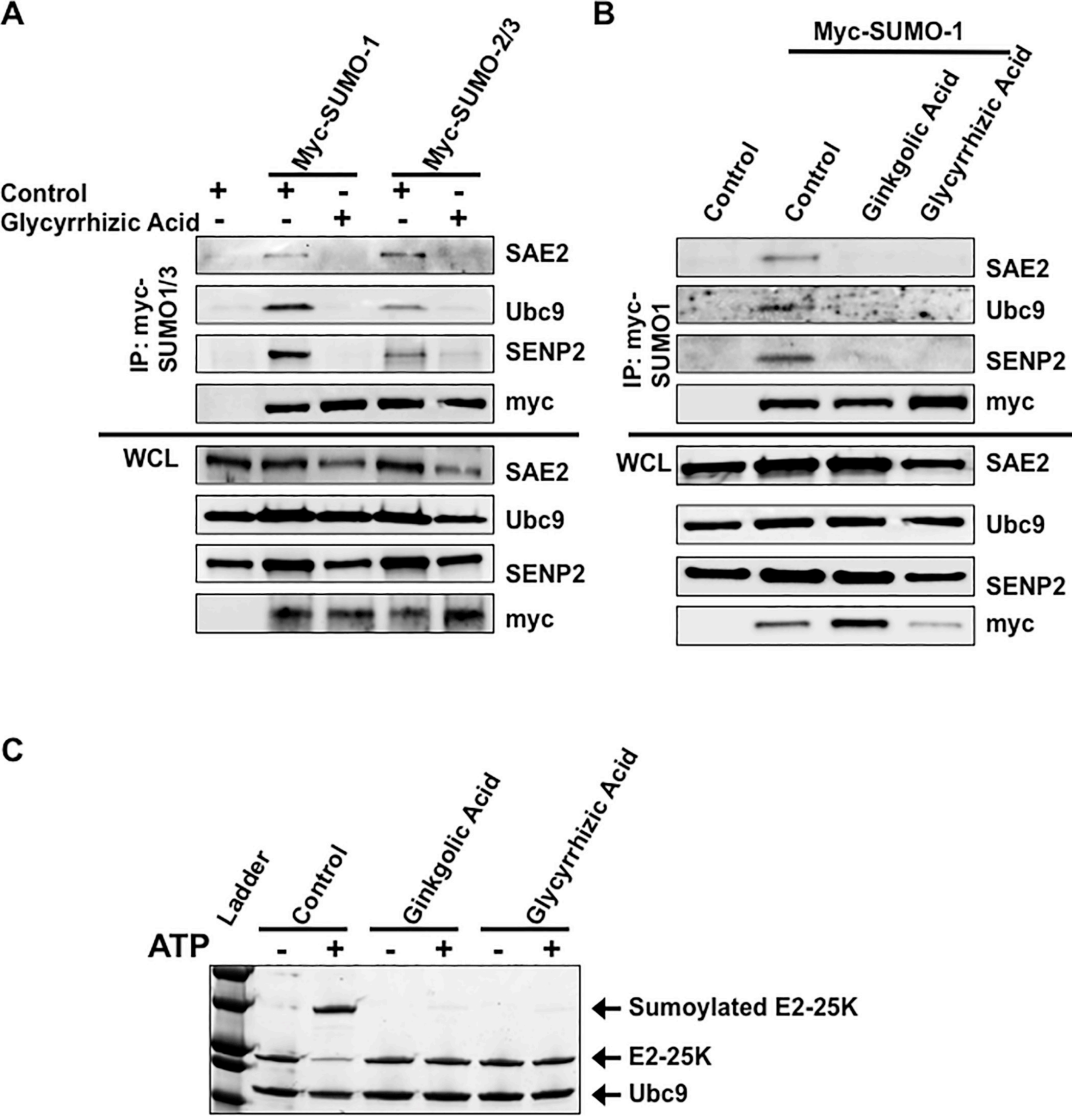
Glycyrrhizic acid inhibited SUMO from interacting with the SUMO machinery and was sufficient to inhibit sumoylation processes *in vitro*. HEK 293 cells were transfected with SAE1/2, Ubc9, SENP2, and myc-SUMO-1 or myc-SUMO-3 expression constructs and treated with A) the vehicle control or glycyrrhizic acid and B) the vehicle control, ginkgolic acid, or glycyrrhizic acid. Native immunoprecipitations were performed to pull-down all myc-SUMO-interacting proteins. Western blot analyses were performed to detect SAE1, Ubc9, and SENP2 in the immunoprecipitants and whole cell lysates (WCL). C) Reactions of purified E2-25K, a ubiquitin-conjugating E3 enzyme that is a known target for sumoylation, SUMO-activating enzyme (SAE1/2), and SUMO-conjugating enzyme (Ubc9) were incubated in the presence or absence of ATP, which is required for protein sumoylation. Reactions were treated with the vehicle control, ginkgolic acid, or glycyrrhizic acid. Following incubation, reactions were denatured, separated by SDS-PAGE, and bands were visualized with a Coomassie Blue stain.

To better understand the mechanism by which glycyrrhizic acid targets sumoylation processes, the effect of the extract on the interaction of the SUMO machinery with SUMO was investigated (Fig 4A). Native immunoprecipitations were performed to pull-down all proteins interacting with myc-tagged-SUMO-1 and myc-tagged-SUMO-2/3. Results showed that the SUMO-activating enzyme subunit 2 (SAE2), the SUMO-conjugating enzyme (Ubc9), and a SUMO-protease (SENP2) all interacted with SUMO-1 or SUMO-2/3 in control-treated cell. These interactions were lost when cells were treated with glycyrrhizic acid, which suggests that glycyrrhizic acid treatment results in loss of the SUMO/SUMO-machinery interaction.

Ginkgolic acid is known to bind to the SUMO-activating enzyme (SAE1/2) and impair it from interacting with and activating the mature SUMO [23,24], so we compared the effect of glycyrrhizic acid on the interaction between SUMO and SAE2, Ubc9, and SENP2 to the effect of ginkgolic acid on these protein-protein interactions (Fig 4B). As expected, in control samples, the interaction of SAE2, Ubc9, and SENP2 with myc-SUMO-1 was detected. Treatment of cells with either glycyrrhizic acid or ginkgolic acid resulted in the lack of SAE2, Ubc9, or SENP2 being pulled-down with the tagged-SUMO-1. These data confirm that glycyrrhizic acid inhibits sumoylation processes by inhibiting SUMO from interacting with the SUMO machinery. In addition, findings suggest that glycyrrhizic acid may function similarly to ginkgolic acid by inhibiting the first step of the sumoylation process (the SUMO/SAE interaction).

Next, the ability of glycyrrhizic acid to specifically target sumoylation processes was evaluated. Using an *in vitro* sumoylation assay, where a known target for sumoylation (E2-25K, a class II ubiquitin-conjugating E3 enzyme) is incubated with free SUMO-1, the SUMO-activating enzyme (SAE1/SAE2), and the SUMO-conjugating enzyme (Ubc9) in the presence or absence of ATP, which is required for protein sumoylation. Reactions were treated with the vehicle control (DMSO), ginkgolic acid (25 uM), or glycyrrhizic acid (2 mM). Results showed that sumoylated E2-25K was detected in control-treated reactions containing ATP (Fig 4C). Consistent with a previous report [76–78], treatment of ATP-containing reactions with ginkgolic acid abrogated the sumoylation of E2-25K. Similarly, glycyrrhizic acid-treatment of ATP-containing reactions also inhibited the sumoylation of E2-25K. Because no other proteins or pathways were present in the *in vitro* assay, we propose that glycyrrhizic acid could specifically inhibit and was sufficient to inhibit sumoylation processes.

### Glycyrrhizic acid targeted the maintenance of EBV latency

**Fig 5:**
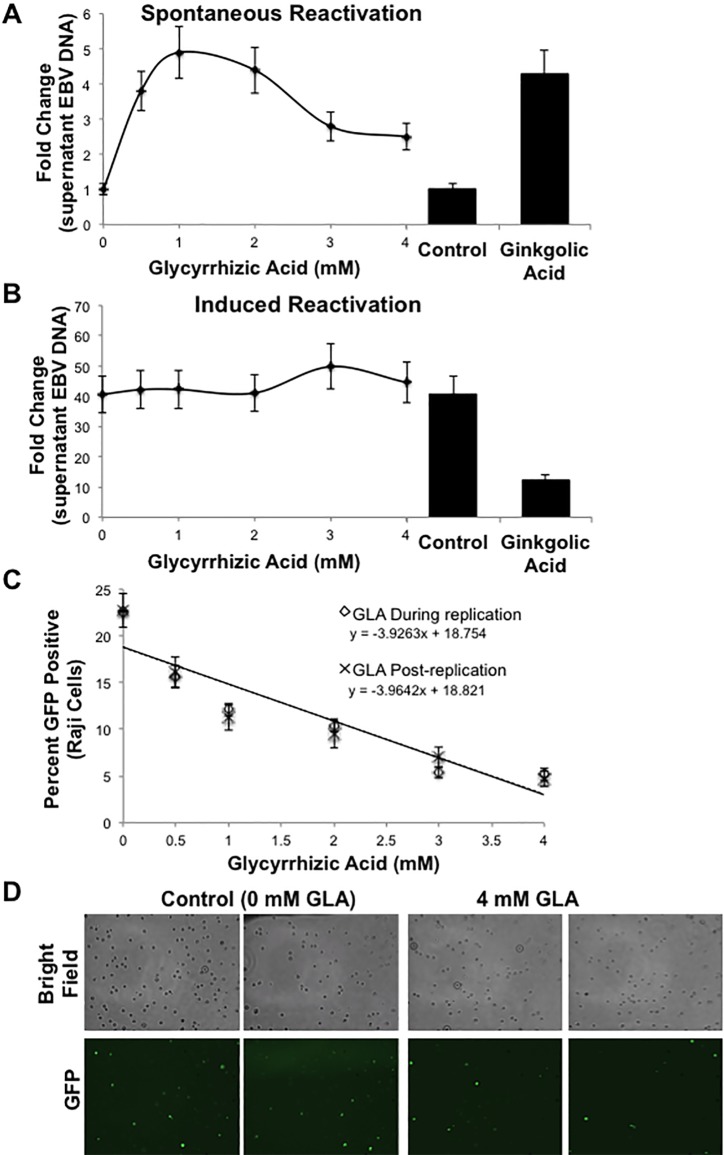
Glycyrrhizic acid treatment targets the maintenance of EBV latency. **A)** HEK 293 EBV BAC cells were treated with DMSO, ginkgolic acid, or graduated amounts of glycyrrhizic acid. Supernatant fluids were collected 72 hours post-treatment, DNase-resistant encapsidated virion associated DNA harvested, and real time PCR performed to quantitate EBV DNA levels and the spontaneous reactivation of the virus. Results are shown as the means ± the standard deviation of experiments performed in triplicate. **B)** HEK 293 EBV BAC cells were transfected with a ZTA-expression construct for the induced reactivation of EBV/ 24 hours post-transfection, cells were treated with DMSO, ginkgolic acid, or graduated amounts of glycyrrhizic acid. Supernatant fluids were collected 72 hours post-transfection, DNase-resistant encapsidated virion associated DNA harvested, and real time PCR performed to quantitate EBV DNA levels. Results are shown as the means ± the standard deviation of experiments performed in triplicate. **C)** EBV reactivation was induced in HEK 293 EBV BAC cells with transfection of ZTA-expression constructs. Cells were treated with graduated doses of glycyrrhizic acid (or the vehicle control) 24 hours post-transfection (GLA During replication) or collected supernatant fluids were treated with graduated doses of glycyrrhizic acid (GLA Post-replication). Collected supernatant fluids were used to superinfect Raji cells. The percent GFP-positive Raji cells were determined by immunofluorescence microscopy. Results are shown as the means ± the standard deviation of samples run in triplicate and experiments performed in triplicate. **D)** Representative images of random fields of view for control- and glycyrrhizic acid-treated cells.

We previously identified a function for LMP1 in the maintenance of EBV latency due to the sumoylation of the transcriptional repressor KRAB-associated protein-1 (KAP1), which binds to and represses the lytic EBV promoters [25]. Here, we suggest that glycyrrhizic acid can inhibit cellular sumoylation processes, so the effect of the extract in the maintenance of EBV latency was examined. HEK 293 cells stably expressing the EBV WT bacterial artificial chromosome (HEK 293 EBV BAC from Dr. Wolfgang Hammerschmidt [61]) were treated with DMSO (vehicle Control), ginkgolic acid, or graduated doses of glycyrrhizic acid to quantitate the spontaneous reactivation of EBV (Fig 5A). Confirming our previous report, ginkgolic acid treatment resulted in a four-fold increase in the spontaneous reactivation of EBV [25]. Similarly, treatment with glycyrrhizic acid resulted in significant (P < 0.05) increases in EBV DNA levels when compared to control-treated cells. These findings suggest that treatment with either glycyrrhizic acid or ginkgolic acid results in low levels of spontaneous reactivation.

To determine the effect of glycyrrhizic acid on lytic viral replication, HEK 293 EBV BAC cells were transfected with an EBV ZTA-expression constructs to induce reactivation (Fig 5B). 24 hours post-transfection, cells were treated with DMSO (control), ginkgolic acid, or graduated doses of glycyrrhizic acid. The fold change in EBV DNA levels (relative to non-induced reactivation control cells) were determined 48 hours post-treatment. Data revealed a 40-fold increase in EBV DNA levels in cells following induced reactivation (Control) when compared with cells where reactivation was not induced. Treatment of cells with glycyrrhizic acid did not alter EBV DNA levels following an induced reactivation; however, treatment with ginkgolic acid significantly (P < 0.05) inhibited the induced reactivation of EBV. These data suggest that ginkgolic acid can inhibit viral replication following induced reactivation but glycyrrhizic acid does not affect lytic replication, which is consistent with earlier reports [46,47].

### The presence of glycyrrhizic acid decreased EBV penetrance

Because glycyrrhizic acid did not affect EBV DNA levels following induced reactivation, the ability of the produced virus to super-infect Raji cells was examined [68]. HEK 293 EBV WT cells were transfected with an EBV ZTA-expression constructs to induce reactivation of EBV, and cells were treated with the vehicle control or graduated doses of glycyrrhizic acid. 48 hours post-treatment, supernatant fluids were used to super-infect Raji cells. 72 hours post-infection, the percent GFP-positive Raji cells (Fig 5C; GLA During replication) was determined. Findings showed that an average of 20–25% of Raji cells were super-infected with new virus, which is consistent with our previous work [25]. Supernatant fluids from glycyrrhizic acid-treated cells resulted in a significant (P < 0.05) decreases in Raji cell super-infection (Fig 5C and 5D). To evaluate whether the presence of glycyrrhizic acid in the supernatant fluids was sufficient to inhibit the super-infection of the Raji cells, supernatant fluids were collected from HEK 293 EBV WT cells following induced reactivation. The supernatant fluids were treated with graduated doses of glycyrrhizic acid, and the ability of the virus in these supernatant fluids to super-infect Raji cells was determined after 72 hours (Fig 5C; GLA Post-replication). Findings showed a dose-dependent decrease in Raji cell super-infection with post-replication glycyrrhizic acid treatment. In fact, no differences were detected when comparing results from supernatant fluids treated during EBV replication with supernatant fluids treated post-EBV replication. These results suggest that regardless to when treated, the presence of glycyrrhizic acid decreases the ability of EBV to infect new cells, which coincides with previous reports [46,47].

Together, these finding identify that glycyrrhizic acid can specifically inhibit sumoylation processes in B-cells, including cells latently infected with EBV. While glycyrrhizic acid treatment can result in low levels of viral reactivation, it does not affect lytic replication. However, the presence of glycyrrhizic acid inhibits the ability of any produced virus from infecting additional cells. Therefore, our proposal that treatment with glycyrrhizic acid may be beneficial in the treatment of EBV-associated malignancies, as well as other diseases in which sumoylation processes are up-regulated, remains.

## Discussion

One proposed therapeutic target for cancer is the sumoylation process [2,21]. Earlier, we identified a novel function for LMP1, in the dysregulation of cellular sumoylation processes during EBV latency [25,62,64], and our recent work documented that SUMO levels are increased in LMP1-positive lymphoma tissues [79]. Therefore, identifying mechanisms by which sumoylation processes can be inhibited may aid the treatment of LMP1-associated malignancies. Our current findings imply that glycyrrhizic acid, a triterpene from licorice root [32,33], inhibits cellular sumoylation processes and can be used to inhibit the growth of EBV-transformed lymphoblastoid cell lines and induce apoptosis. This is the first report using glycyrrhizic acid to target sumoylation processes. In addition, these findings provide further support for the function of sumoylation in the maintenance of latency [25] and verify that glycyrrhizic acid can inhibit EBV infection, which has been proposed due to inhibition of viral penetration [46,47]

Here, we document that glycyrrhizic acid inhibits cellular sumoylation processes in multiple cell lines. To rule out the possibility that the detected decreases in global sumoylation levels was due to decreased expression of the SUMO machinery, we did examine the levels of the SUMO-activating enzyme (SAE1/SAE2), the SUMO-conjugating enzyme (Ubc9), one of the few identified SUMO-E3 ligases, and SUMO proteases (SENPs) following glycyrrhizic acid treatment. SAE1/2, Ubc9, and SENP2 levels were not affected by the botanical extract. The maturation (by SENPs), activation (by SAE1/2), and conjugation (by Ubc9) of SUMO to the target protein are essential steps during the sumoylation process. Therefore, we can conclude that the mechanism by which glycyrrhizic acid inhibits cellular sumoylation processes was not by decreasing the levels of the essential SUMO machinery. Interestingly, higher levels of glycyrrhizic acid treatment did result in decreased levels of the two examined SUMO ligases (PIAS1 and RanBP2). The SUMO E3 ligases are thought to act as an adaptor between the Ubc9-SUMO intermediate and the target protein, conferring specificity towards the target proteins [72,80]. It is possible that glycyrrhizic acid-mediated decrease in levels of sumoylated proteins may be specific to the targets of PIAS1 and RanBP2. However, it would be advantageous to elucidate if the extract has similar effects on other SUMO ligases.

Treatment of cells with glycyrrhizic acid did not affect cellular ubiquitination processes, suggesting some selectivity in targeting cellular processes. *In vitro* sumoylation assays showed that glycyrrhizic acid was sufficient to inhibit protein sumoylation, which leads us to propose that the extract can specifically target the sumoylation process. However, glycyrrhizic acid does not exclusively target cellular sumoylation processes. Instead multiple signaling pathways are modulated by this extract [81,82], which could provide additional advantages when used to modulate the multitude of signal transduction pathways induced by the principal viral oncoprotein.

Immunoprecipitation studies revealed that like ginkgolic acid, glycyrrhizic acid treatment results in loss of the interaction between the SUMO machinery and SUMO. Ginkgolic acid is known to target the first step of the sumoylation process [23]. Because of the observed similarities when comparing glycyrrhizic acid treatment with ginkgolic acid treatment (Fig 4B and 4C), we propose that glycyrrhizic acid inhibits the SUMO-activating enzyme from interacting with SUMO, which leads to the subsequent decreases in SUMO from interacting with Ubc9 and SENP2. While the immunoprecipitation experiments were performed in HEK 293 cells due to their increased ability to be transiently transfected, we predict similar results would be observed in any other cell line.

Our earlier work identified that LMP1 induced the sumoylation of KAP1 [25], a well-characterized transcriptional co-repressor [83]. KAP1 also binds to and represses EBV oriLyt and the immediate early promoters [25]. Others have shown that the SUMO E3 ligase PIAS1 can aid the maintenance of EBV latency [84]. Specifically, caspase-3, -6, and -8 cleave PIAS1, decreasing PIAS1 levels and increasing the spontaneous reactivation of EBV [84]. Caspase activation has also been implicated in the spontaneous reactivation a different γ-herpesvirus, Kaposi’s sarcoma-associated herpes virus [85,86]. We did detect that higher doses of glycyrrhizic acid treatment resulted in increased activation of caspase-3, which corresponded with decreased PIAS1 levels; however it did not coincide with higher levels of spontaneous reactivation of the virus. Regardless, the changes in sumoylation levels, the increased activation of the caspases, and the decrease in PIAS1 levels could all contribute to the weakening of LMP1-mediated maintenance of latency, which we now show occurs following the treatment of latently infected cells with glycyrrhizic acid. However, as shown here, which coincides with previous reports [46,47], the presence of glycyrrhizic acid significantly (P < 0.05) inhibits the ability of any produced virus to infect new cells. Specifically, the presence of glycyrrhizic acid in the environment, added during an induced reactivation or after an induced reactivation, did significantly inhibit any produced virus from infecting new cells. Therefore, even low levels of spontaneous reactivation would not be detrimental to the host. However, it does remain to be determined if glycyrrhizic acid inhibits the infectivity of virus produced from cells.

Consistent with published data, our findings suggest that glycyrrhizic acid does not affect EBV levels following induced reactivation [46,47]. Interestingly, induced reactivation was significantly inhibited following ginkgolic acid treatment. While we previously have focused on sumoylation processes during latent EBV infection and spontaneous reactivation [25,62–64], others have investigated functions for sumoylation processes during lytic replication [87–97]. It has been proposed that EBV manipulates sumoylation processes during its lytic cycle in order to provide favorable conditions for optimal replication [95]. SUMO-modified proteins accumulate late during lytic replication [96], in part due to EBV miR-BHRF1-1-mediated decreased levels of the SUMO-targeted ubiquitin ligase RNF4 [96]. Reconstitution of RNF4 levels coincide with reduced expression of early and late EBV proteins and impaired virus release [96]. In addition, four lytic proteins (SM/EB2, BGLF2, BMRF1, and BVRF2) have been shown to globally upregulate SUMO levels when expressed in cells [97]. These reports, along with our finding that ginkgolic acid significantly inhibited induced reactivation, suggest a function for sumoylation processes during lytic replication. However, treatment of cells with glycyrrhizic acid did not affect the induced reactivation of EBV, which raised the question of why these differing results were detected. It is possible that the targeting of other cellular processes by ginkgolic acid that were not inhibited by treatment with glycyrrhizic acid was sufficient to decrease EBV DNA levels following induced reactivation. Therefore, in the future, it would be advantageous to elucidate a function for EBV-mediated increased protein sumoylation during lytic EBV infection.

For the past two decades, glycyrrhizic acid has been used clinically in China and Japan, with satisfactory therapeutic effects [98]. It has been confirmed to be safe and non-toxic [99], and it has inhibitory effects on many cancers, including leukemia, gliomas, colon cancer, and lung cancer [100–111]. Here we show that glycyrrhizic acid has inhibitory effects on EBV-transformed LCLs, which mimic EBV-associated lymphoproliferative diseases. While low doses of glycyrrhizic acid did not affect LCL growth or death, levels as low as 2 mM inhibited LCL growth and started to promote low levels of cleaved caspase 3 and PARP, which resulted in apoptosis. Previous reports used 2.4 mM glycyrrhizic acid and found that it diminished growth of other EBV-positive cell lines (Raji and P3HR1) [46,47]. Therefore, we propose that glycyrrhizic acid would have an inhibitory effect on EBV-associated lymphoproliferative diseases by decreasing proliferation with minimal cell death.

Glycyrrhizic acid and its derivatives have been shown to have a relative lack of toxicity at the cellular level all while inhibiting new EBV infections [47], which suggest the extract may be an efficient and safe treatment for EBV infections. Treatment of latently infected cells with glycyrrhizic acid induced low levels of viral reactivation, which would likely increase in patients undergoing radiation and chemotherapy. We show the presence of glycyrrhizic acid results in the decreased propensity of the virus to be able to infect other cells. Therefore, increases in EBV DNA would not necessarily be detrimental to the host. In fact, glycyrrhizic acid treatment could inhibit the penetration of virus that results from chemotherapy-induced EBV reactivation.

In addition to EBV reactivation, the multi-faceted chemotherapy regime for EBV-associated lymphomas, especially in immunocompromised individuals, often results in liver toxicity [112] or even reactivation of Hepatitis C virus (HCV) or Hepatitis B virus (HBV) [113,114]. Glycyrrhizic acid is currently being used in Asia to inhibit liver fibrosis, steatosis, and necrosis, inhibit the reactivation of HCV and HBV [32,33,35,36], and promote cell regeneration [34,113,114]. It is known to reduce liver toxicity that results from chemotherapy [115–118], even in patients diagnosed with diffuse large B-cell lymphomas and receiving CHOP (cyclophosphamide, vincristine, doxorubicin, and prednisone) therapy [119]. Furthermore, glycyrrhizic acid can aid the intracellular delivery of other administered drugs [102,120–123]. Therefore, treatment with glycyrrhizic acid may be even more beneficial in the treatment of EBV-associated lymphomas than just its ability to inhibit cellular sumoylation processes.

The licorice root also contains flavonoids (quercetin and isoliquiritigenin) [124], which too have anti-cancer and anti-inflammatory properties [125–128]. While nothing is known of the effects of isoliquiritigenin on EBV latency, quercetin was shown to reduce EBV latency [129], inhibit EBV infection in EBV-associated gastric carcinoma cell lines [129], and have anti-cancer effects in *in vivo* xenograft animal models for EBV-positive gastric carcinomas [130]. Consequently, it is likely that other components of the licorice root may also be beneficial to modulating the EBV life-cycle, possibly through regulation of post-translational modifications.

In summary, we propose that during latent EBV infection, LMP1 dysregulates sumoylation processes, resulting in increased protein sumoylation, which may aid tumorigenesis. Glycyrrhizic acid can inhibit sumoylation processes in LMP1-expressing, EBV-transformed lymphoblastoid cell lines, blocking proliferation, increasing cell death, inducing low levels of viral reactivation, and impeding the infection of new cells by the produced virus. Taken together with the numerous therapeutic effects of glycyrrhizic acid, these findings identify a novel pathway targeted by the botanical extract and identify a novel mechanism, by which EBV-associated lymphoproliferative diseases could possibly be treated.
